# Vitalizing Community for Health Promotion Against Modifiable Risk Factors of Noncommunicable Diseases (V-CaN) in Rural Central India: Protocol for a Hybrid Type II Implementation Effectiveness Trial

**DOI:** 10.2196/42450

**Published:** 2023-09-29

**Authors:** Anuj Mundra, Ashwini Kalantri, Arjunkumar Jakasania, Harshal Sathe, Abhishek Raut, Chetna Maliye, Pramod Bahulekar, Ajay Dawale, Rameshwar J Paradkar, Sakshi Siriah, Satish Kumar, Subodh S Gupta, Bishan Garg

**Affiliations:** 1 Department of Community Medicine Mahatma Gandhi Institute of Medical Sciences Kasturba Health Society Wardha India; 2 Department of Psychiatry Mahatma Gandhi Institute of Medical Sciences Wardha India; 3 District Health Office Zilla Parishad Nagpur India; 4 District Health Office Zilla Parishad Wardha India; 5 Department of Biochemistry Mahatma Gandhi Institute of Medical Sciences Wardha India

**Keywords:** noncommunicable diseases, action research, implementation research, community-based participatory research, salutogenesis, primary prevention

## Abstract

**Background:**

Low- and middle-income countries are facing the emerging burden of chronic noncommunicable diseases (NCDs). Apart from loss of human lives and premature deaths, NCDs result in huge costs for treatment to individuals and the health system. Although NCDs develop in later life, the risk factors begin at an early age. The key to the control of the global epidemic of NCDs is primary prevention based on comprehensive community-based programs.

**Objective:**

This study aims to develop, implement, and evaluate the effect of a participatory health promotion initiative utilizing the existing mechanisms of Village Health Nutrition and Sanitation Committees (VHNSCs), women’s self-help groups (SHGs), and schools on modifiable risk factors for NCDs among young people aged 10-30 years.

**Methods:**

The proposed type II hybrid effectiveness implementation cluster randomized field trial will be conducted in the catchment area of 4 primary health centers (PHCs) in Wardha district, India, comprising 100 villages with a population of 144,000. Each PHC will be randomly allocated to one of the 3 intervention arms or the control arm. The 3-intervention arm PHCs will utilize a unique strategy with either VHNSC or SHG members or school students as change agents for health action against common modifiable NCD risk factors. This study will be implemented in 3 phases from January 2022 to December 2024. First, the preparatory phase for baseline assessments includes anthropometry, behavioral and biochemical risk factors for NCDs, and participatory development of the health promotion intervention modules. Second, the implementation phase will focus on capacity building of the change agents and implementation of the participatory health promotion initiative. The implementation will include organization of community-based events, 6-monthly participatory assessment of change, and preparation of a sustainability and exit plan toward the end of this phase. Third, the evaluation phase will consist of studying the effectiveness of each intervention strategy in the reduction of risk factor prevalence at the population level.

**Results:**

We will assess 12,000 (3000 in each arm) randomly selected individuals for behavioral risk factors and 1600 (400 in each arm) individuals for biochemical risk factors during baseline as well as endline assessments. Difference in differences, ANOVA or multivariate analysis of covariance, and regression analysis will be performed to assess the effectiveness of the interventions. Qualitative methods such as focus group discussions and stories of change will be documented and analyzed using thematic framework analysis. The implementation outcomes will be reported using the PRISM (Practical Robust Implementation and Sustainability Model) RE-AIM (Reach, Effectiveness, Adoption, Implementation, Maintenance) framework. The results are expected to be published by mid-2025.

**Conclusions:**

This study will show the magnitude of risk factors for NCDs, its determinants, feasibility, effectiveness of community-based interventions, and health promotion models for NCD prevention.

**Trial Registration:**

Clinical Trials Registration India CTRI/2020/10/028700; https://ctri.nic.in/Clinicaltrials/showallp.php?mid1=47597&EncHid=&userName=V-CaN

**International Registered Report Identifier (IRRID):**

DERR1-10.2196/42450

## Introduction

### Background

Low- and middle-income countries such as India are facing the emerging burden of chronic noncommunicable diseases (NCDs) while still struggling with the unfinished agenda of improving maternal and child health, nutrition, and infectious disease spread [1]. The burden of cardiovascular diseases, chronic respiratory diseases, diabetes, cancer, and mental disorders is predicted to increase significantly in the next 25 years [1]. NCDs are estimated to account for about 75% of all deaths in India by 2030 [1]. Cancer deaths are projected to double from 2004 to 2030, and cardiovascular disease deaths are predicted to increase from 2.7 million in 2004 to 4 million in 2030 [1]. Apart from high costs to the health system, NCDs result in high out-of-pocket expenditure. The overall economic burden of NCDs in India is estimated to amount to approximately 5%-10% of the gross domestic productivity, thus imposing huge social and economic costs, impeding not only health improvements but also economic progress and social development of the country [2]. Evidence suggests that NCDs disproportionately impact people at younger ages and are increasingly afflicting the poorer segments of the society [2,3].

The National Family Health Survey has shown that throughout India, over 10% of the population aged 15 years and older have elevated blood sugar levels and over 20% of the population have elevated blood pressure levels [4,5]. Various studies [6-9] have highlighted the need of early interventions for the primary and secondary prevention of NCD-related risk factors. For instance, a study in Central India reported that the prevalence of hypertension was 5.75% among adolescents, which highlights the need for blood pressure and nutritional monitoring among adolescents [6]. Overweight or obesity has been reported in 4.3% of schoolchildren in Wardha, with a dietary deficiency in 57% of the adolescent girls [7]. Metabolic syndrome (clustering of abdominal obesity, hypertension, disturbed glucose metabolism, and dyslipidemia) has been reported in 9.3% of the rural adult population in India [8]. A recent review from low- and middle-income countries highlights the role of community-based interventions through health-promotion initiatives in reducing the risk factors related to NCDs. Such interventions significantly reduce the risks ranging from lifestyle and diet modifications, smoking, alcohol, etc [9].

The key to the control of the global epidemic of NCDs is primary prevention based on comprehensive community-based population-wide programs. There is a common agreement that community empowerment is crucial for the success of such programs, and partnerships among different stakeholders are required to provide effective pathways for the design and delivery of NCD prevention and control programs. Although India counts on different national programs to control NCDs, the gaps in implementing these programs and a clear strategy of what works and how to implement it at the local level is lacking. Notably, health promotion is a common component in all these programs [2,9,10]. Indian states such as Tamil Nadu and Kerala have piloted efforts around NCD prevention and control through various health promotion activities [11]. Although some community-based interventions have been conducted in India, there is a great variation in terms of the components of the interventions and the target population. However, to have a population-level impact and to reach communities in the periphery, the health promotion component of the NCD programs needs to be adapted to the local context [2,9].

Few studies [12-17] focusing on lifestyle intervention programs at the community level have shown considerable benefits in terms of promoting health education through healthy diets, increasing physical activity, and screening for diabetes prevention and control [12]. A community-based study in Gujarat targeting adults for the prevention and control of diabetes, consisting of health education sessions provided by trained community health workers, led to improved knowledge of diabetes and cardiovascular diseases and a reduction in the overall blood pressure in the studied population [13]. A community-based program in India for NCD prevention and control in Ballabgarh district was evaluated by Krishnan et al [14]. Volunteers and schoolteachers were trained in addition to communication campaigns, camps, and reorientation of health services at the community level. The intervention led to better management of NCDs at health facilities [14].

Several school-based health education programs in India have successfully reduced the rates of both tobacco experimentation and offer of tobacco by peers [15,16]. School-based educational programs can significantly influence children’s behavior in inculcating healthy lifestyle practices. A school-based intervention called MARG (medical education for children or adolescents for realistic prevention of obesity and diabetes and for healthy aging) showed a significant increase in the intake of healthy foods, decrease in the intake of fried and fatty energy-dense foods, and increase in the physical activity and time spent on outdoor games along with improvements in glycemic parameters and lipid profiles [17]. The MYTRI (Mobilizing Youth for Tobacco-Related Initiatives in India; 2002-2007) project consisted of randomized controlled intervention trials that established the effectiveness of school health interventions in reducing tobacco use among Indian adolescents by reducing current tobacco use and their future intentions to use tobacco and by enhancing their health advocacy skills [16].

Several studies [18-22] have also emphasized the potential of self-help groups (SHGs) in improving health-related outcomes at the population level, especially in terms of knowledge, attitude, and practice of healthy behaviors for NCDs as well as other health-related conditions. VHNSCs were constituted under the National Rural Health Mission in India to enable community participation in health-related activities, formulate village health action plans, and implement and monitor the same in the villages. The VHNSC is responsible for the health of the villagers, and they are mandated to undertake preventive, promotive, and curative health activities at the village level [23].

In this study, we aim to develop, implement, and evaluate the effects of a participatory health promotion initiative utilizing the existing mechanisms of VHNSC, women’s SHGs, and schools on behavioral as well as biochemical modifiable risk factors for NCDs (obesity or overweight, tobacco use, stress, unhealthy diet, physical inactivity, harmful use of alcohol, high blood pressure, high blood sugar, and increased blood cholesterol) among young people in the age group of 10-30 years.

### Rationale of This Study

Randomized clinical trials have documented that lifestyle changes can prevent NCDs [24]. However, there is limited evidence, particularly in India, whether such strategies are applicable at the community level, that is, in a real-life setting. The emphasis of our proposed study is to strengthen the primary prevention of NCDs, which is often neglected under the current national health programs. Our proposed study will utilize a health promotion approach as indicated in the Ottawa Charter for Health Promotion [25] and will make efforts to empower and enable young people aged 10-30 years to exercise control and take care of their own health. Our study will focus on young people aged 10-30 years, as this age group is not covered under the National Programme for Prevention & Control of Non-Communicable Diseases (NP-NCD), and it is during this phase of life that NCD-related risk factors begin, which become habits during later years and thus become difficult to change and lead to chronic diseases. Apart from focusing on the commonly emphasized risk factors of tobacco, alcohol, and physical activity, this study will include locally feasible and acceptable strategies to reduce stress, which often is not accounted for in NCD prevention. This study will be implemented in collaboration with the district health system, utilizing the existing mechanisms of Village Health Nutrition and Sanitation Committee (VHNSC), women’s SHGs, and schools by using the principles of participatory development and therefore much more likely to be acceptable to the community and sustainable eventually. The NP-NCD currently emphasizes early diagnosis and prompt treatment and at times tends to overlook the primary prevention. Our study, which is the first of its kind in central India for health promotion against NCD risk factors among young people aged 10-30 years, will help strengthen the primary prevention component under the NP-NCD. The proposed study has the potential of producing a community-based health promotion model for the prevention of NCDs through the life cycle approach, and thus, has every chance of leading directly to improvements in population health.

## Methods

### Study Design

The proposed study will be a type II hybrid effectiveness implementation cluster randomized field trial that will be implemented in a rural area of Wardha district of Maharashtra, India, which also is a part of the field practice area of the Department of Community Medicine, Mahatma Gandhi Institute of Medical Sciences, India. This study will be performed in the catchment areas of 4 primary health centers (PHCs) of Wardha block, namely, Anji, Kharangana, Talegaon, and Waifad. As per the census of 2011, there are around 100 villages in this study area with a population of approximately 144,000 people. However, the census towns (as per census 2011) in the study area will be excluded [26]. This study will be performed from January 2022 to December 2024.

### Randomization

The proposed study will have 4 arms: 3 intervention arms and 1 control arm. The unit of randomization would be PHCs and each of the 4 PHCs will be randomly allocated using a simple randomization technique through the lottery method to 1 of the 3 intervention arms or to the control arm. The primary strategy in this study would be health promotion by creating change agents. The 3 intervention arms would differ in the source of change agents. The 3 interventions in this proposed study would be with (1) school students as change agents, (2) VHNSC members as change agents, and (3) women’s SHG members as change agents.

### Study Implementation Plan

**Figure 1:**
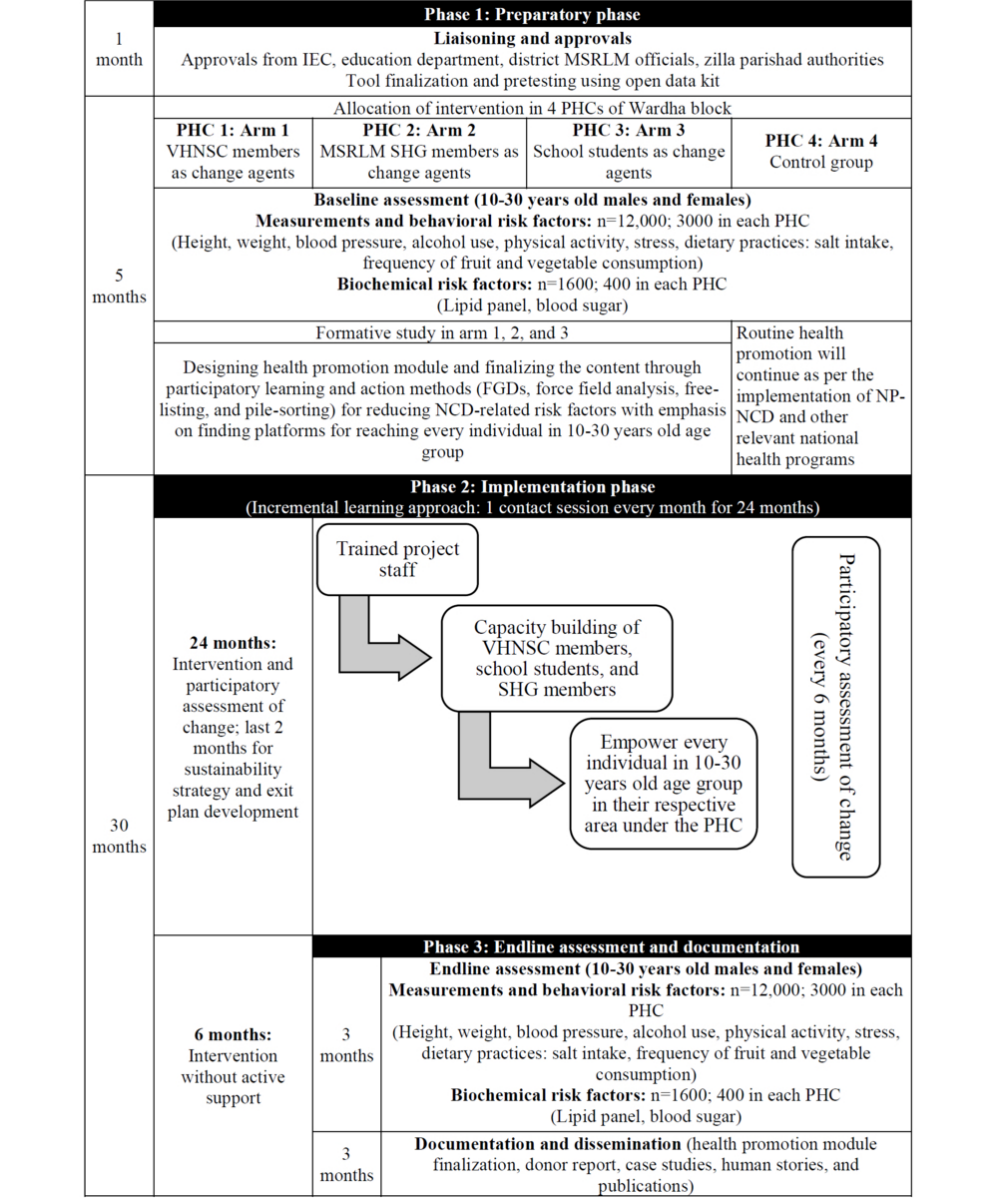
Study flowchart showing the phases and respective activities. FGD: focus group discussion; IEC: institutional ethics committee; MSRLM: Maharashtra State Rural Livelihood Mission; NCD: noncommunicable disease; NP-NCD: National Programme for Prevention & Control of Non-Communicable Diseases; PHC: primary health center; SHG: self-help group; VHNSC: Village Health Nutrition and Sanitation Committee.

This study will be implemented in collaboration with the district health system. As per randomization of the study arms, the existing mechanisms of VHNSC, women’s SHGs, and schools would be utilized for study implementation by using the principles of participatory development in a phased manner as depicted in Figure 1 and as described below.

#### Phase 1: Preparatory Phase (6 Months)

The preparatory phase will include liaising and seeking administrative approvals, staff recruitment, baseline data collection, and a formative study for developing the health promotion module.

##### Liaising and Seeking Administrative Approvals (First Month)

The preparatory phase will include seeking approval from the institutional ethics committee and concerned district authorities from health, education, zilla parishad, and livelihood mission departments. Piloting of data collection tools and planning for baseline data collection will be performed.

##### Baseline Data Collection (Second Month to Fourth Month)

A baseline assessment of the prevalence of NCD-related modifiable risk factors, that is, obesity, addiction, stress, and dietary practices, including salt consumption, physical activity, and hypertension or prehypertension will be conducted using the STEPS approach developed by the World Health Organization (WHO) [27]. The study questionnaires will include sociodemographic details, NCD risk factor survey, stress questionnaire, anthropometry, and blood pressure measurements. The details of these tools are given in a later section. Sample size was estimated separately for behavioral and biochemical risk factors by using G*power software (version 3.1; Institute for Experimental Psychology) for behavioral as well as biochemical risk factors.

##### Sample Size for Behavioral Risk Factors

We hypothesize that our interventions would reduce the modifiable behavioral risk factors for NCDs by 25% over a period of 3 years. With an assumption of the lowest prevalence of NCD-related risk factors at 13% [6-8,28,29] to detect the aforesaid difference, with 95% CIs, power of 80%, intracluster correlation coefficient of 2, an approximate sample of 12,000 participants, that is, 3000 in each arm, was calculated. Sample size was estimated using the lowest risk factor prevalence, as it gives the maximum possible sample size. We will utilize stratified random sampling (stratified by gender) to select the study participants. The sampling frame will be prepared from the already existing health and demographic surveillance system, which is established by the Department of Community Medicine, Mahatma Gandhi Institute of Medical Sciences, in 5 PHCs (including the study PHCs) covering around 113 villages and a population of about 120,000 people. The health and demographic surveillance system is updated every 6-12 months, and data from the most recent round will be used for the abovementioned purpose.

##### Sample Size for Biochemical Risk Factors

An approximate sample of 1600 participants, that is, 400 in each arm, has been estimated. This sample size will help to detect an effect size of 10% mean difference from baseline with 90% power, 95% CIs, assuming 10% sample wastage. These 1600 participants would be a randomly selected subset of the 12,000 respondents (as specified for assessing the change in behavioral risk factors). The random sampling will be performed using the “sample_n” function of “dplyr” package in R software (version 4.1.2; R Foundation for Statistical Computing).

##### Inclusion and Exclusion Criteria

All individuals in the age group of 10-30 years, as selected through the sampling procedure described earlier who or whose parents or guardians give written informed consent, will be included in this study for evaluation of the intervention. Those experiencing severe illness or chronic diseases will be excluded from the study since their behavioral and biochemical risk factors may be influenced by their diseased status.

##### Formative Study for Developing the Health Promotion Module (Second to Sixth Month)

Formative research using qualitative methods and participatory learning and action techniques will be performed, which will help us in understanding the current practices pertaining to NCD-related risk factors and help us identify the possible opportunities and mechanisms to implement the intervention. Focus group discussions, force-field analysis, free-listing, and pile-sorting will be performed to understand the community perceptions on NCD-related risk factors, their prevention, and control. Based on the findings of the formative study, the health promotion module will be developed. These modules will be central to the participatory health promotive interventions to be implemented in the next phase. The health promotion module will have incorporated monthly session plans that will be implemented through monthly contact sessions in an incremental learning approach. The health promotion module will also emphasize deciding strategies for reaching every young person in the age group of 10-30 years and its delivery mechanisms. The strength of this strategy will be that the concerned stakeholders will be able to identify and implement initiatives that are acceptable and feasible to them (eg, stress mediation strategies, local strategies for increasing consumption of locally available and easy to access nutritious food, promotion of physical activity).

#### Phase 2: Intervention Phase (30 Months)

##### Design

**Figure 2:**
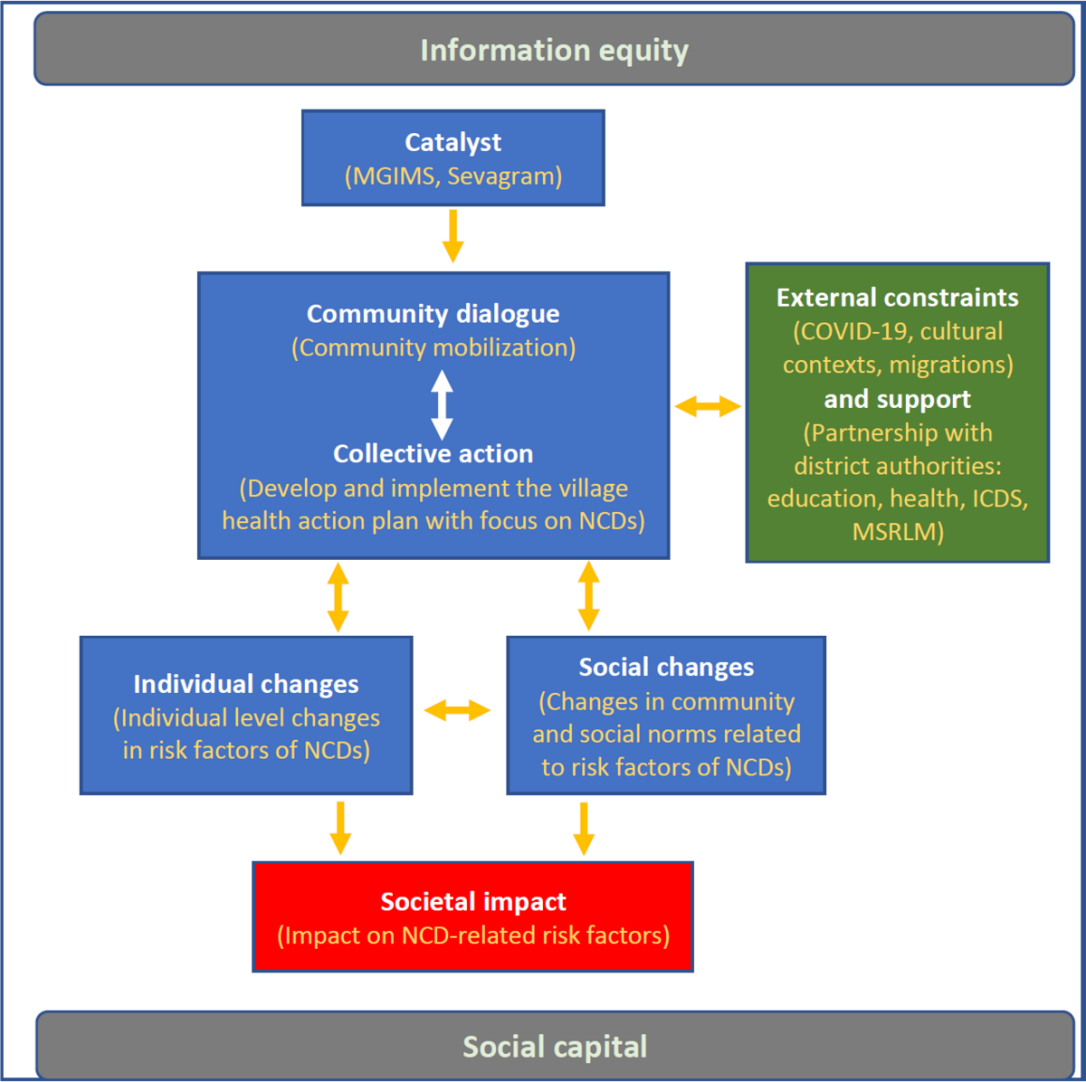
Adaptation of the integrated model of communication for social change in this study (adapted from Figueroa et al [30], which is published under Creative Commons Attribution 4.0 International License [31]). ICDS: Integrated Child Development Services; MGIMS: Mahatma Gandhi Institute of Medical Sciences; MSRLM: Maharashtra State Rural Livelihood Mission; NCD: noncommunicable disease.

The intervention phase will include the implementation of the participatory health promotion initiative based on the integrated model of communication for social change (Figure 2) [30]. Of the total duration of 30 months, 24 months will be active intervention through the study and 6 months will be the intervention without active support to assess the sustainability of the intervention. VHNSCs, although mandated to undertake preventive, promotive, and curative activities at the village level since many years, now need further capacity building to undertake such activities. The capacity of the existing VHNSCs will be used to implement the village health plan against NCDs in coordination with various community-based organizations such as SHGs, farmer groups, and adolescent groups. As discussed earlier, the health promotion module will be implemented in the community through VHNSCs, school students, and SHG members as change agents. The population in all the 4 arms will continue to receive routine care through the existing government programs for NCDs. However, no additional activities will be conducted in the control arm in this study.

##### Formation of Vitalizing Community for Health Promotion Against Modifiable Risk Factor NCD (V-CaN) Clubs

The key implementation strategies through these change agents in the intervention arms will be the formation of the Vitalizing Community for Health Promotion Against Modifiable Risk Factors of NCD (V-CaN) clubs for developing and implementing an NCD-specific health action plan for their own villages, undertaking various community-based events for health promotion, celebration of V-CaN days for spreading awareness, and creation of a social norm. The formation of these V-CaN clubs will be facilitated by the study team, and the change agents in their respective arms will be encouraged to form V-CaN clubs in their village with representation from all concerned stakeholders from that village to help disseminate and promote the learnings of the participatory health promotion module.

These V-CaN clubs will focus on preparing NCD-specific health action for themselves. The V-CaN clubs will also give emphasis on relieving stress through locally acceptable and feasible stress meditation strategies. A monthly meeting of these V-CaN clubs will be conducted to review monthly activities and ensure prompt health actions. As specified earlier, there will be no additional activities (V-CaN club formation) in the control arm; however, the population in the control arm will continue to receive routine care through the existing government program, that is, NP-NCD.

##### NCD-Specific Health Action Plan

The V-CaN club members will be expected to make a health plan against NCDs to take the learning from the monthly sessions forward. The health plan will focus on reaching every person in the age group of 10-30 years in their area. It will also include activities that they will undertake during the next 2 years for bringing about behavior change in young people. The plan will focus on identifying and implementing initiatives that are acceptable and feasible to the local community for motivating the community to indulge in pro-health behavior as described above.

##### Organizing V-CaN Days

A V-CaN day (Melawa) will be organized in all the intervention villages and schools once in every 6 months with the objective to facilitate the behavior change process in the participants to (1) give up tobacco and alcohol consumption habits, if any, (2) eat a proper balanced diet, and (3) indulge in physical activity every week. The activities on the V-CaN day will be arranged in collaboration with village level health functionaries, namely, VHNSCs, ASHA (Accredited Social Health Activist), and AWW (Anganwadi worker). The effort will be to emphasize the harmful effects of the NCD risk factors by using modes such as exhibitions, role plays, demonstrations, and participatory games. Efforts will be taken to maximize the participation in these V-CaN day celebrations.

##### Sustainability Strategy and Exit Plan

**Figure 3:**
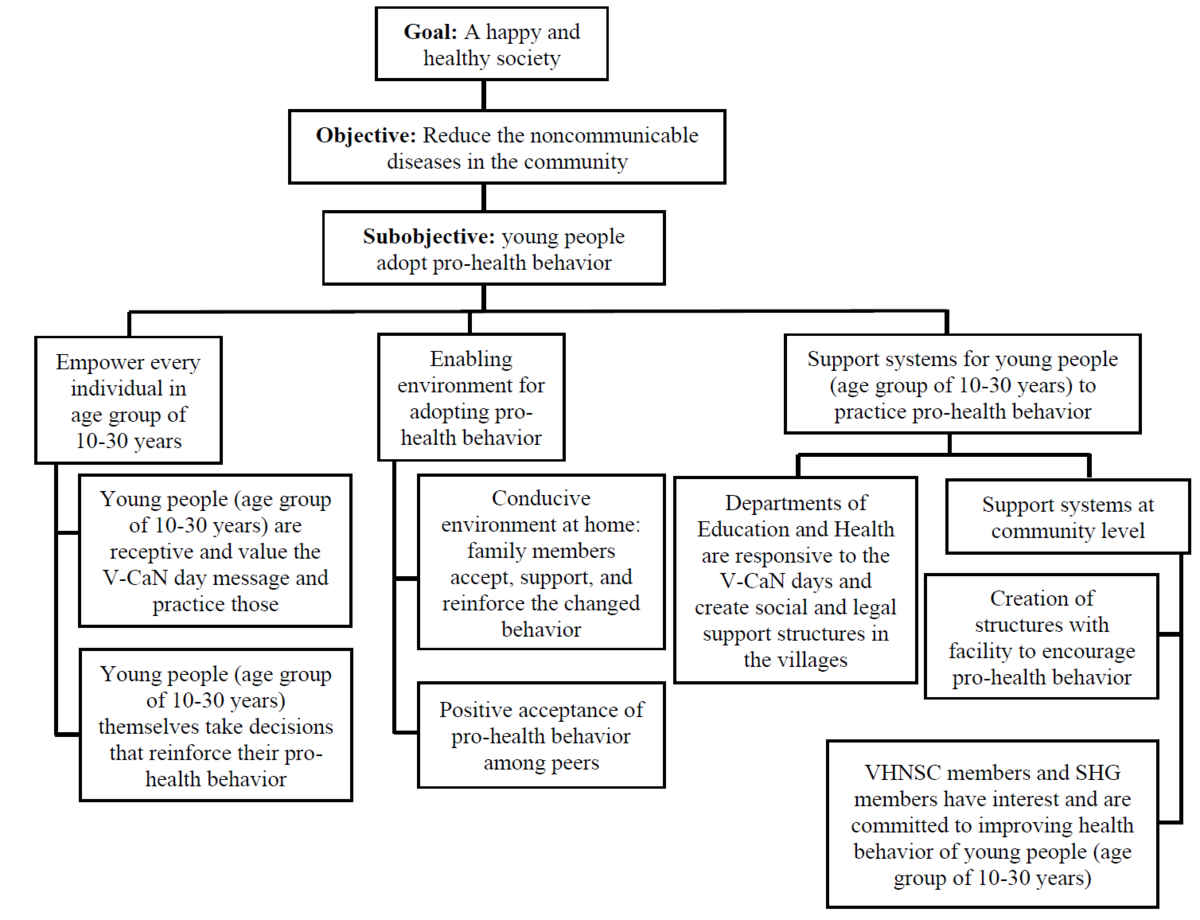
Theory of change for this study. SHG: self-help group; V-CaN: Vitalizing Community for Health Promotion Against Modifiable Risk Factors of Noncommunicable Diseases; VHNSC: Village Health Nutrition and Sanitation Committee.

An exit plan will be prepared in consultation with the V-CaN clubs regarding how to sustain the initiative after the implementation phase of the study. Each V-CaN club will be facilitated to prepare a plan for mobilizing resources for continuing the activities of V-CaN club. In the last 6 months of the implementation phase of the study, the activities will continue without any active support from the study staff. The sustenance of activities for the next 6 months will be monitored and documented through the study and will be a proxy indicator of the sustainability of the intervention. Factors affecting sustainability will be studied through doer (villages or schools that are able to sustain) and non–doer analyses (villages or schools that are not able to sustain). We have developed a theory of change (Figure 3) and a results framework (Table 1) for the study that will be used for monitoring and evaluation (process, output, and effect) of the study and documenting the change.

**Table 1:** Results framework for the proposed Vitalizing Community for Health Promotion Against Modifiable Risk Factors of Noncommunicable Diseases (V-CaN) study.

Questions	Answers	Key assumptions
What is the problem you are trying to solve?	Enabling every young person to adopt a pro-health behavior	Quality and longevity of the intervention
Who is your key audience?	All young people aged 10-30 years	Young people are receptive and value the V-CaN^a^ day messages and practice them
What is your entry point for reaching your key audience?	VHNSC^b^ members, school students, MSRLM^c^ SHG^d^ members	Health and education departments are responsive to V-CaN days and give due priority to them VHNSC members, school students, and MSRLM SHG members have interest and are committed to improving the health behavior of young people
What steps are needed to bring about the change?	Receiving administrative approvals Necessary supplies and logistics Training of trainers (supervisors) Training and capacity building of VHNSC members, school students, and MSRLM SHG members Reaching all young people aged 10-30 years through identified approaches	VHNSC members, school students, and MSRLM SHG members are able to tide over the social norms or restrictions and conduct or participate in V-CaN day activities
What is the measurable effect of your work?	Impact Obesity or overweight Prehypertension Hypertension Pro-health behavior of all young people Effect Decreased prevalence of prehypertension or hypertension Decreased prevalence of overweight or obesity Increased frequency and consumption of fruits and vegetables Increased level of physical activity Decreased stress levels Decreased tobacco use Decreased alcohol use Decreased salt intake Output V-CaN days held (number conducted or number planned) V-CaN clubs formed Proportion of young persons (10-30 years) reached	Master trainers have interest and are committed to improving the health behavior of young people Good quality in-system trainings are planned and conducted Master trainers are able to transfer the skills to VHNSC members, school students, and MSRLM SHG members
What are the wider benefits of your work?	Decreased prevalence of noncommunicable diseases	Young people have the necessary family and social support to engage in pro-health behavior
What is the long-term change you see as your goal?	A healthy and happy society	Stakeholders are young children and their parents, panchayat raj institution members, VHNSC members, school students, and MSRLM SHG members

^a^V-CaN: Vitalizing Community for Health Promotion Against Modifiable Risk Factors of Noncommunicable Diseases.

^b^VHNSC: Village Health Nutrition and Sanitation Committee.

^c^MSRLM: Maharashtra State Rural Livelihood Mission.

^d^SHG: self-help group.

##### Participatory Assessment of Change

Concurrent evaluations using the participatory method of web (spider) diagram will be performed every 6 months to assess behavior change among the young people.

#### Phase 3: Endline Assessment and Documentation (6 months)

Endline surveys and assessments for the effectiveness of health intervention measures will be performed in both the intervention and control groups. Case studies and human stories of change will be documented.

### Data Collection and Statistical Analysis Plan

#### Data Collection Tools

The data collection instrument will be designed based on the WHO-STEPwise tool for NCD risk factor surveillance during baseline as well as endline assessments [27]. The WHO-STEPwise approach to the surveillance of NCD risk factors uses a standard survey instrument and a methodology that can be adapted to different country resource settings. It will be suitably modified to meet the objectives of this study and will be pilot tested. Data will be collected on Android-based tablets by using a survey solution platform developed by the World Bank through individual surveys. Anthropometry, physical measurements, and biochemical assessments will also be conducted in young people in the age group of 10-30 years. The data collection tools will consist of (1) baseline demographic information about individuals in households (age, gender, education, occupation, socioeconomic status, etc); (2) health behaviors consisting of tobacco use in smoked and smokeless form, alcohol consumption, dietary pattern (consumption of fruits, vegetables, salt, etc), and physical activity; (3) stress measurements of the study participants by using the perceived stress scale-10 that has already been used and validated in India [32,33]; (4) physical measurements, which will include measurements of height, weight, and blood pressure; and (5) biochemical assessments, which will include fasting blood sugar and lipid profile.

Operational definitions of health behaviors and procedures for physical measurements and biochemical assessments will be as per the standard procedures in the WHO-STEPwise tool for the NCD risk factor surveillance. Height and weight will be measured as described in the WHO STEPwise surveillance module. For blood pressure measurements, all the readings will be recorded by trained personnel to reduce the interobserver variation. Training in all relevant techniques will be given, including care for avoiding expectation error and digit preference. The forearm will be kept at the level of the heart. The sphygmomanometer will also be kept at the level of the heart. Two readings of blood pressure will be taken: one at the beginning of the assessment and the second at midway in the assessment. If the first 2 readings differ by more than 5 mm Hg, an additional reading will be taken and the average of the 3 readings will be noted. For blood sugar and lipid profile, 5 mL of fasting venous blood sample will be collected and transferred to the central laboratory of the institute for further processing and reporting [34]. As specified in the methodology earlier, all the measurements would be performed on 12,000 participants, except for biochemical assessments, which will be performed for a random subset of 1600 participants from among the larger cohort of 12,000 participants. However, a separate random sampling will be performed as specified earlier for the endline assessment as well.

#### Statistical Analysis Plan

The primary outcome variable for this study would be the prevalence of various modifiable NCD risk factors as compared to baseline. Descriptive analysis will be performed using proportions with 95% CIs. For the quantitative information, difference in differences analysis using *Z*-test for differences between proportions of independent samples and ANOVA or multivariate analysis of covariance will be used to find out the effectiveness of the intervention between the intervention and the control groups. Here, the 3 interventions will be evaluated independently to demonstrate the feasibility and efficacy of each of the 3 intervention modalities. In addition, multivariate regression will be performed for finding the determinants of the risk factors, and exploratory factor analysis will be performed to explain the observed variances between intervention and control groups. For the qualitative data, thematic content analysis will be performed.

### Quality Assurance

A 3-member project steering committee will be formed for technical guidance and overall quality assurance of the study. The committee will consist of a senior public health professional, a member of the district administration, and a health communications expert. Standard operating procedures will be developed for measurements of blood pressure, height, and weight. Standard and calibrated ISO-marked nonmercury automated sphygmomanometers will used throughout the study to minimize the instrumental error. Height and weight will be measured using standard calibrated electronic weighing scales and stadiometer. Similarly, standard operating procedures will be developed for blood collection and transport of samples for fasting blood sugar and lipid profile estimation. Blood sugar and lipid profile estimation will be performed in the National Accreditation Board for Testing and Calibration Laboratories–accredited central laboratory of the institute. Approximately 5% backcheck of the filled forms will be performed by study assistants for ensuring the quality of data collection. Supportive supervision checklists will be prepared for concurrent quality monitoring for monthly meetings of V-CaN clubs and V-CaN days.

### Ethics Approval

This study has been registered with Clinical Trials Registration India prospectively on October 28, 2020. Ethics approval has been obtained from the institutional ethics committee, Mahatma Gandhi Institute of Medical Sciences, before the start of the trial vide letter MGIMS/IEC/COMMED/79/2020. A written informed consent will be taken from all adults before enrolling in this study. For schoolchildren and adolescents, written consent will be taken from their parents and verbal assent from the participants. Treatment of study participants diagnosed with hypertension during screening will be ensured at the respective PHC. If needed, a referral to the appropriate higher center will be given to the study participants. Study participants will be interviewed and examined in private; women or adolescent girls will be examined in the presence of another female. The information in the study records will be kept strictly confidential. The identity of the study participants will be kept confidential by assigning them a unique identification number. Anonymized data would be used for scientific publications. Data quality will be checked before analysis.

## Results

**Figure 4:**
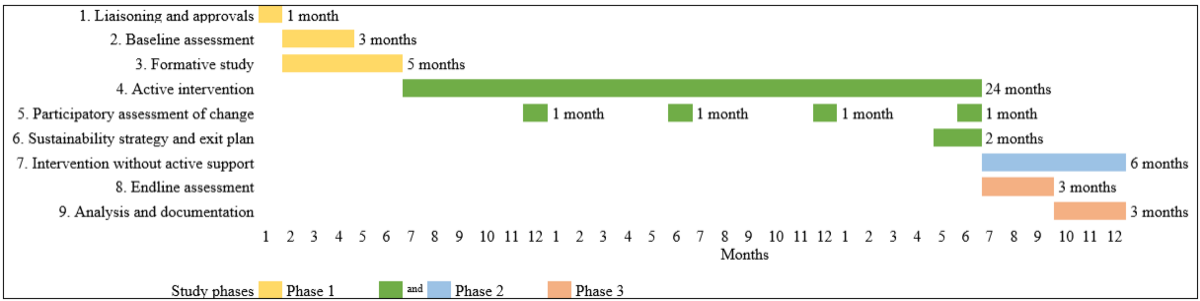
Timelines for the proposed study.

This study will be performed over a course of 3 years, as described in Figure 4, and the results are expected to be ready to be published or disseminated by mid-2025. Our findings will be disseminated through scientific publications in peer-reviewed journals, academic seminars, national and international conferences, and administrative stakeholders at the district, state, and national levels. Special dissemination strategies will be conducted at community and school levels through local-level gatherings and meetings.

## Discussion

Our study is one of its kind trying to focus on reducing the exposure to risk factors related to NCDs in young people in the age group of 10-30 years. The findings of this study will provide us with evidence based on exposure prevention and lifestyle modification at a stage when exposures become habits and subsequently the lifestyle.

### Expected Outcomes

This study will help to find out the magnitude of the problem of risk factors for NCDs in rural areas, its determinants, and the feasibility and effectiveness of a community-based trial. This study will also provide insights into the positive influences and barriers for behavioral change communication. The findings of this study will help us to develop a community-based health promotion model for the prevention of NCDs through the life cycle approach. Thus, the proposed research has every chance of leading directly to improvements in population health. We will use the PRISM (Practical Robust Implementation and Sustainability Model) RE-AIM (Reach, Effectiveness, Adoption, Implementation, Maintenance) framework to assess the implementation outcomes as outlined in Tables 2 and 3 so as to facilitate its translation into policy and practice [35,36].

**Table 2:** Description of the PRISM (Practical Robust Implementation and Sustainability Model) contextual domains and elements incorporated in the study.

PRISM^a^ domains		Elements of PRISM domains
**Program (intervention)**		
	Organizational perspective	Readiness of the institute, government administration, and investigators Coordination and collaboration between MGIMS^b^ and district authorities of health, MSRLM^c^, and education departments as well as interdepartmental collaboration Strong evidence base of behavioral risk factors targeted in the study Estimated high burden of NCDs^d^ in future Usability and adaptability of the community mobilization process Mechanism for addressing barriers of study staff
	Individual (beneficiary) perspective	Community-centered interventions Community empowerment to adopt healthy behavior Addressing barriers of beneficiaries in participation and adoption of behavior change during V-CaN^e^ days/monthly V-CaN meetings Feedback of results to be shared
**Recipients**		
	Organizational characteristics	Schools, VHNSCs^f^, MSRLM SHGs^g^—implementation climate across the 3 intervention arms Developing and implementing village health action plans through V-CaN clubs Trainings given under the study Keeping the group motivated as a whole to work toward healthy behavior Existing community norms Time management for study-related activities along with primary objectives of the organization Decision-making
	Individual (beneficiary) characteristics	Keeping the group members motivated to work toward healthy behavior Knowledge and beliefs of individuals Competing interests of individuals
Implementation and sustainability infrastructure		Universally existing infrastructure of schools, VHNSCs, and SHG networks Dedicated study team for study duration and V-CaN clubs for future sustainability Participatory approach for preparing health promotion modules and planning of interventions Scope of flexibility/creativity as per the village health action plan prepared under the study Exclusively dedicated time duration for studying sustainability 6 monthly participatory assessments of change and program evaluation at the end of the study
External environment		Existing national program for NCDs (NP-NCD^h^) Competing health agenda and priorities Migrations Behavioral/cultural acceptability for pro-health behavior Community resource mobilization

^a^PRISM: Practical Robust Implementation and Sustainability Model.

^b^MGIMS: Mahatma Gandhi Institute of Medical Sciences.

^c^MSRLM: Maharashtra State Rural Livelihood Mission.

^d^NCD: noncommunicable disease.

^e^V-CaN: Vitalizing Community for Health Promotion Against Modifiable Risk Factors of Noncommunicable Diseases.

^f^VHNSC: Village Health Nutrition and Sanitation Committee.

^g^SHG: self-help group.

^h^NP-NCD: National Programme for Prevention & Control of Non-Communicable Diseases.

**Table 3:** Description of the RE-AIM (Reach, Effectiveness, Adoption, Implementation, Maintenance) critical outcome indicators to be assessed.

RE-AIM^a^ dimensions		RE-AIM indicators
Reach		Characteristics of participants, that is, students, SHG^b^ members, VHNSCs^c^ (eg, age, gender, education) and comparison of characteristics across the 3 arms Participation rates (as proportion of all eligible/approached) Dropout rates
**Effectiveness**		
	Primary	Change in the prevalence of risk factors related to NCDs^d^ Feasibility of each intervention for reduction of risk factor prevalence
	Secondary	Characteristics of individuals who comply with the behavior change versus noncompliers Change in the prevalence of obese/overweight individuals, patients with hypertension or prehypertension, patients with diabetes or prediabetes Change in the mean blood pressure/blood sugar/serum cholesterol levels of population
Adoption		Proportion of V-CaN^e^ club monthly meetings held/V-CaN days celebrated out of committee Reasons for missed V-CaN club meetings/V-CaN day celebrations (comparison across the 3 arms)
Implementation		Fidelity of intervention sessions (trainings, V-CaN meetings, V-CaN days) Content delivery and satisfaction of beneficiaries Best practices, innovations, and improvisation in the 3 arms
Maintenance		Sustainability and exit plan Proportion of villages delivering the interventions during the last 6 months of the study period without active support of the study team Enabling and inhibiting factors for maintenance/discontinuation and adaptation

^a^RE-AIM: Reach, Effectiveness, Adoption, Implementation, Maintenance.

^b^SHG: self-help group.

^c^VHNSC: Village Health Nutrition and Sanitation Committee.

^d^NCD: noncommunicable disease.

^e^V-CaN: Vitalizing Community for Health Promotion Against Modifiable Risk Factors of Noncommunicable Diseases.

### Future Plans Based on Expected Outcomes

This study will be a value addition to strengthen the primary prevention component under the NP-NCD and Rashtriya Kishor Swasthya Karyakram, as it is going to be implemented in collaboration with the district health system, utilizing the existing mechanisms of VHNSC, community-based organizations, and frontline health workers. If proven effective, the possibility to implement the health promotion modules in the control PHC will also be explored at the end of the study. Further advocacy with the administration will be performed for scaling up of the intervention.

### Strengths and Limitations of This Study

This cluster randomized study will provide rich evidence regarding prevention of common modifiable risk factors against NCDs. Moreover, outcome evaluation is performed in a very large sample, thereby minimizing the possibility of random errors in the results. Simple random sampling will ensure minimal selection bias. The inbuilt concept of community mobilization and preparation of an exit plan may also lead to sustenance of the program, thereby ensuring the perceived benefits to reach the people even beyond the study duration. External generalizability of this study will be limited only for rural areas with similar profiles and not for urban or tribal areas.

## Abbreviations

ASHA: Accredited Social Health Activist
AWW: Anganwadi worker
MARG: medical education for children or adolescents for realistic prevention of obesity and diabetes and for healthy aging
MYTRI: Mobilizing Youth for Tobacco-Related Initiatives in India
NCD: noncommunicable disease
NP-NCD: National Programme for Prevention & Control of Non-Communicable Diseases
PHC: primary health center
PRISM RE-AIM: Practical Robust Implementation and Sustainability Model Reach, Effectiveness, Adoption, Implementation, Maintenance
SHG: self-help group
V-CaN: Vitalizing Community for Health Promotion Against Modifiable Risk Factors of Noncommunicable Diseases
VHNSC: Village Health Nutrition and Sanitation Committee
WHO: World Health Organization

## Data Availability

After the completion of the proposed study, the findings will be published in a peer-reviewed journal. The data from this study may be available with the authors and will be accessible on request from the corresponding author.
